# HIV treatment 2020: what will it look like?

**DOI:** 10.7448/IAS.17.4.19528

**Published:** 2014-11-02

**Authors:** Roy Gulick

**Affiliations:** Department of Medicine, Weill Medical College of Cornell University, New York, USA

## Abstract

Currently there are 28 approved antiretroviral drugs in six mechanistic classes, and recommended first-line regimens are highly potent, well tolerated, and as convenient as one pill, once-a-day. How will HIV treatment change by 2020? Over the next few years, we are likely to see potent 2-drug regimens tested head-to-head with standard three-drug regimens, and some of these will likely become standard-of-care. Newer agents with novel drug resistance profiles (e.g. doravirine, an NNRTI) or new mechanisms of action (e.g. BMS 663068, a CD4 attachment inhibitor) will provide virologic activity in patients with drug-resistant viral strains. Comparative studies of current and newer agents such as the investigational prodrug of tenofovir (TAF) will help define less toxic regimens. We will see additional convenient co-formulations developed; with them, we are likely to have second- and even third-line regimens administered one pill, once-daily. Long-acting injectable investigational formulations currently in clinical trials such as rilpivirine LA (administered monthly) and cabotegravir (administered quarterly), and others (including combinations of these agents) could provide additional convenient treatment options. Other novel formulations (e.g. patches, implants, rings) and combinations of antiretrovirals with other kinds of medications (e.g. contraceptives) may be developed and tested. In the developing world, we will see increasing numbers of patients taking potent, well-tolerated convenient first-line and subsequent regimens with the goal of “20 by 20” – 20 million treated people by 2020. Generic formulations of antiretroviral drugs, including combinations, will be increasingly available and used worldwide. With the current appreciation that inflammation and immune activation play an important role in the natural history of treated HIV infection, anti-inflammatory agents will be tested and may supplement (or even be co-formulated with) standard antiretroviral regimens. Recognizing our progress to date, these and other innovations will further improve HIV therapy by 2020.

